# Supplementary material for: Peer and lay health work for people experiencing homelessness: A scoping review
Source: PLOS Glob Public Health. 2024 Jun 24;4(6):e0003332. doi: 10.1371/journal.pgph.0003332 (PMC11195950; doi:10.1371/journal.pgph.0003332)
Supplement: S1 Text — (DOCX) [file pgph.0003332.s002.docx]

MEDLINE Search Strategy

1. Exp ill-housed persons/
2. “People experience*homelessness”.kf,tw.
3. (Unsheltered p* or unstable hous*).kf,tw.
4. ((Homeless or ill-housed or street or shelterless or unhoused) adj1 (person or persons or shelter or people)).kf,tw.
5. 1 or 2 or 3 or 4
6. Exp Community Health Workers/
7. (community adj3 health health work*).kf,tw.
8. (peer adj3 support work*).kf,tw.
9. (lay adj3 volunteer*).kf,tw.
10. (health adj3 ambass*).kf,tw.
11. (lived adj3 experience*).kf,tw.
12. (outreach adj3 worker*).kf,tw.
13. Exp Peer Group/
14. 6 or 7 or 8 or 9 or 10 or 11 or 12 or 13
15. (inhibit* or barrier*).kf,tw.
16. (enabl* or facilitator*).kf,tw.
17. (challenge* or obstacle*).kf,tw.
18. (success adj3 factor*).kf,tw.
19. (inhibit* adj3 factor*).kf,tw.
20. (enabl* adj3 factor*).kf,tw.
21. “effective*”.kf,tw.
22. Engagement.kf,tw.
23. “perception*”.kf,tw.
24. “experience*”.kf,tw.
25. 15 or 16 or 17 or 18 or 19 or 20 or 21 or 22 or 23 or 24
26. 5 and 14 and 25
27. Limit 26 to English
