# Supplementary material for: Peer and lay health work for people experiencing homelessness: A scoping review
Source: PLOS Glob Public Health. 2024 Jun 24;4(6):e0003332. doi: 10.1371/journal.pgph.0003332 (PMC11195950; doi:10.1371/journal.pgph.0003332)
Supplement: Attachment — Submitted filename: Response to Reviewers.docx [file pgph.0003332.s003.docx]

Jessica Mangan

School of Public Health and Social Policy

University of Victoria

April 1, 2024

Hanna Landenmark

Staff Editor

PLOS Global Public Health

Manuscript PGPH-D-24-00025

Response to Reviewers

Dear Hanna Landenmark,

Thank you for allowing us to submit a revised draft of the manuscript “Peer and lay health work for people experiencing homelessness: A scoping review” for consideration by PLOS Global Public Health. We appreciate your work and the time in handling our manuscript. We are grateful for the constructive comments and suggestions from the reviewers. We have incorporated most of the suggestions and revised our manuscript accordingly. Our responses to each comment are below. Please refer to the revised manuscript with tracked changes for the completed revisions. We hope that our revisions have addressed the concerns and improved the quality of our paper.

We look forward to hearing from you soon.

**Journal Requirements**

| **Editor Comment** | **Author Response** |
| --- | --- |
| We noticed you have some minor occurrence of overlapping text with the following previous publication(s), which needs to be addressed:  - DOI: 10.1002/cl2.1154 https://www.pathhomekingston.ca/homelessness-in-our-community/ -Stewart, Miriam & Reutter, Linda & Letourneau, Nicole & Makwarimba, Edward. (2009). A Support Intervention to Promote Health and Coping Among Homeless Youths. The Canadian journal of nursing research = Revue canadienne de recherche en sciences infirmières. 41. 55-77.  In your revision ensure you cite all your sources (including your own works), and quote or rephrase any duplicated text outside the methods section. Further consideration is dependent on these concerns being addressed. | DOI: 10.1002/cl2.1154 is cited as reference [39]; Moledina et al., 2021. It was cited on pages 15, 16, 19 and 22.  <https://www.pathhomekingston.ca/homelessness-in-our-community/> uses stats on the number of people in Canada and the United States experiencing homelessness. We retrieved those numbers from references [4 (Employment and Social Development Canada, 2019), 5 (Gaetz et al., 2016), and 6 (U.S. Department of Housing and Urban Development, 2022)] which we believe were the original sources from the government of Canada and the government of the United States. The website you provided states those stats without any reference from where they got the data from.  Stewart et al., 2009 is cited as reference [48]. It was cited on pages 14, 15, 16, 20, 21, and 22.  Please let us know if you identify anything else not properly cited so we can address it immediately. |

**Reviewers’ Comments to the Authors**

**Reviewer 1**

| **Reviewer comment** | **Author response** |
| --- | --- |
| Title - could the title be more informative? At the moment it tells the reader that it's about peer workers, homelessness and it's a scoping review. I wonder whether including the outcome, by saying, Impact of peer worker programs on service access for people experiencing homelessness? | Thank you for your comment. We have revised the title to be more informative:  ‘Enablers and inhibitors of effective peer and lay health work for people experiencing homelessness: A scoping review’. |
| Definition of peer and lay workers - I recommend removing 'are individuals without professional training' because they may have education in other fields, just not homelessness or social work. | Thank you for your comment. We have removed that line from the sentence (page 3, line 22). |
| Question/objectives - could this be slightly more clear by using the PiCO approach - population, intervention, context, outcome. The aim of the review is a little busy, perhaps the aim could be to explore the impact of lay and peer worker programs.... The concept explored didnt quite match the aim, because the effectivenens of peer workers is stated here and it isnt in the aim. The outcome is about factors that hinder or facilitate, so perhaps this could all be captured in the aim. | Thank you for your comment. We presented the review question in PCC format (population, concept, context) using the PRISMA-ScR guidelines.  Population: people experiencing homelessness (PEH)  Concept: factors that facilitate or hinder effective peer and lay health worker programs within the homeless sector  Context: PEH participating in any type of peer or lay health worker programs  The concept explored was edited to better capture that the outcome is about factors that hinder or facilitate (page 4, line 14). |
| Information sources - CINAHL not CINHAL. In developing the search strategy, who was it that peer reviewed it? Were these other authors, a research librarian or people with lived experience? I recommend including the search terms in the paper itself. | Thank you for catching that error. The spelling of CINAHL was revised throughout the manuscript.  In developing the search strategy, the lead author drafted the search strategy and refined it in consultation with peer reviewers (the other authors) (page 5, line 13-14).  Thank you for your suggestion. However, the search terms are currently listed in the search strategy in the appendix *‘Supplementary File 2: MEDLINE Search Strategy’* as the list is quite comprehensive that it may overwhelm the flow of the paper. We would be happy to do whatever the reviewer suggested - if the editors prefer. |
| Selection of sources - page 9 - the term 'characteristics of effective peer and lay programs...' can you be more specific and name who is deciding whether they are effective and on what basis? Is it effective in relation to housing or health access, or effective in terms of building rapport? The paragraph on types of study included could perhaps be deleted because the first line of this section states that any study design was included. Why did you dedulicate prior to uploading to COVIDENCE, when COVIDENCE does this automatically? In screening the papers could you provide more detail on the review process in relation to disagreements. Which authors undertook screening, which authors then reviewed disagreements and what was the definition of consensus - was it 75% of the authors agreed? | We have thoroughly revised based on your feedback. We inserted the names of who specifically screened/selected papers and how disagreements were resolved in the selection of sources paragraph (page 6, line 19-25).  The sentence regarding effectiveness was modified – as the concept explored was the factors that facilitate or hinder effective peer and lay health worker programs in the homeless sector (page 6, line 10). In terms of housing, house access, building rapport, etc. – it is dependent on each study.  Thank you for your comment. The paragraph on types of studies was deleted (on page 6).  The sentence about deduplicating prior to uploading to COVIDENCE was deleted – this was an error – COVIDENCE did that for us (page 6, line 19). |
| Data extraction - it is a limitation if only one author extracted data - did anyone check this for accuracy? Was the synthesis of the data also undertaken by one author? This is also a limitation | Correct – only one author completed data extraction and synthesis. This is stated under the limitations paragraph (page 22, line 3). |
| Results - It seemed a bit unusual that literature reviews were included in the included papers of a scoping review. From experience this can make data extraction very complex, and sticking with primary research can be a more streamlined approach. Table 1 - It would be helpful to provide more information regarding the column headings. I wouldn't consider homelessness as a condition, more a circumstance or experience. Homelessness was also an inclusion criteria so I would expect all papers to relate to homelessness, therefore no need for a column. Also, column heading outcome reported, this is not very detailed, what do you mean specifically by social outcomes, or health outcomes, were these improvements in certain conditions, or access to housing. This outcomes column is important as it tells use the potential impact of peer support workers. As a suggestion, perhaps include the title of each paper in the table because that will give the reader a lot more information, and it would also be helpful to understand the study aim too. The column about employment status is not particularly informative either, and perhaps this is something that could be removed from the table and discussed in the narrative instead. From reading the table I really dont get a sense of the papers included. The descriptions of the themes of the studies is informative. There could be slightly more detail, for example, how many studies were from which countries, how many were cohort studies - simply place (n=x) after each statement. | We appreciate your insightful concern. We found the 3 included systematic reviews were very informative to helping identify and develop common themes. However, we have revised the format of table 1 to separate the primary studies and knowledge synthesis to make it clearer (i.e., includes the 3 systematic reviews).  Thank you for your comment. We greatly appreciate your feedback on formatting the tables. We have removed the condition and employment status columns, while adding a column to display the full title of the articles included.  In terms of the column heading outcome reported, it was difficult to be specific in the heading as we were interested in both social and health outcomes (dependent on the focus of the particular study). We greatly appreciate your suggestion about adding the title of each paper in the table as it aided in giving the reader much more information to interpret the rest of the table.  Thank you for your comment. (n=x) was placed after a few statements. Please see page 14, line 5-7 and page 14, line 17. |
| Table 2 is informative. Consider adding some examples e.g., how was stigmatisation an inhibitor - who was experiencing the stigma or from whom was this behaviour perceived? Rather than state 'Poor attendance' as a heading, consider simply stating 'Attendance' otherwise it might be perceived as slightly judgmental. There will be lots of genuine reasons why people dont attend, many of which will be out of the person's control. | Thank you for your comment on Table 2. Additional information was added. For example, it was updated that it was referring to the stigmatization of clients being judged, poorly treated and dismissed by healthcare providers (page 16, line 25). Majority of the table was meant to be very concise and further elaborated on in the results and discussion.  Thank you for the suggestion of reframing ‘poor attendance’ as a heading – it has been updated to just ‘attendance’ (page 20, line 12). |
| Discussion. Please review the areas of text without references. For example, page 25, starting A critical element... the text really needs supporting with references. Likewise the subsequent paragraph. Consider adding a section in the discussion around the future of peer workers and given their impact, ways that we can employ them in the field of homelessness. Do we need policy change, education programs, government funding? | Thank you for pointing that out. ‘A critical element…’ is now supported with references (page 22, line 8).  ‘Peer support work programs…’ (page 22, line 9) is now supported with references.  ‘Additionally, clients benefit from…’ (page 22, line 13) is now supported with references.  ‘Peer support work programs offer a sense of…’ is supported with a reference (page 22, line 15).  The paragraph, ‘peer programs create a safe and inclusive environment…’ is now supported with references (page 22, line 19).  Thank you for that very insight suggestion. We added a section in the discussion regarding the future of peer workers (page 23, line 1). |
| Limitations - only having one person extract data definitely impacts rigour - it is good this is acknowledged, I recognise deleting the statement about the compromise of the rigor. Is it a limitation that there were no RCT's? | Thank you for your comment. A sentence regarding not including RCTs was added to the limitation paragraph (page 23, line 17):  ‘We acknowledge that not including randomized controlled trials may be considered a limitation, as they are considered the gold standard for assessing effectiveness of interventions. Unfortunately, due to this topic being so novel there are very minimal randomized controlled trials available.’ |

**Reviewer 2**

| **Reviewer comment** | **Author response** |
| --- | --- |
| The background section appears to be missing something between “homelessness” paragraph and “scope”. A sentence or two summarising the literature on peer work in homelessness would be beneficial here to give better flow as currently it feels a bit jarring to go from describing challenges associated with homelessness to the enablers of a peer workforce. | Thank you for this insightful feedback. I moved the paragraph ‘definition of peer and lay workers’ from under the methods section to fit between the ‘homelessness’ paragraph and ‘scope’, which seems to give better flow (page 3, line 16).  Another sentence was added to the beginning of ‘scope’ to improve the flow of the paragraph (page 4, line 3). |
| Under questions and objectives you refer to “the participants in this review” – as they aren’t participating in anything – perhaps “population of interest” or similar would better describe this. | Thank you for your comment, we changed the wording of this sentence using your feedback (page 4, line 13). |
| Under Selection of sources of evidence - some of the paragraphs could be simplified (or even deleted) by creating a table with a column for inclusion, and a column for exclusion criteria. | Thank you for this insightful feedback; it was similar to reviewer #1’s feedback. Using both of your feedback on this section, we have deleted the paragraph on the types of studies (on page 6). |
| Last sentence of last para in selection of sources- regarding consensus of conflicts – was this consensus made between the two reviewers? Or was a third author brought in? | The consensus was made between the two reviewers – this paragraph has been updated in the text to be more specific (page 6, line 24). |
| Characteristics table – Few inconsistencies to check, i.e., in the Sydney study they are referred to as advisors and advisers. Sometimes refer to the participants and “youth” and sometimes as “young people. Sometimes “not reported” is followed with a full-stop, but most of the time not. This may be one to check with the editor, but I find having a footnote at the end of the table with all the abbreviations in useful | Thank you for bringing this to our attention. The Sydney study has been edited to, ‘advisers’. Each study utilizes different terminology. We have gone through each row to ensure the language is consistent for each study.  We appreciate your valuable suggestion. A footnote at the end of the table with all of the abbreviations utilized was added (page 13). |
| Populations and personnel – “almost all peer workers had a lived exp of homelessness” – how many? | Thank you for this feedback – the sentence has been revised. N=20 studies had peer workers with lived experience of homelessness (page 14, line 11). |
| Table 2 – I’m hesitant to raise this one as I do find the information in the table very useful! But another way this information could be presented is as a tick box matrix table whereby all the studies are listed in the first column, then each of the types of barriers/enablers are listed in the first row. (i.e., column two might be “trust & relationships” and all studies that discuss that as an enable have a tick). This would make it a bit more user friendly if someone was looking for specific barriers/enablers within peer work in homelessness to find the articles of relevance to them as currently would need to look through the table to try and identify the 21 articles yourself as they aren’t specifically referenced in the paragraph on this. Anyway, please see this as a suggestion only rather than a requirement! | Thank you so much for this extremely thoughtful suggestion. We gave this suggestion a lot of thought and even created a rough draft of the proposed table with tick boxes… Ultimately, we decided to keep the original format of table 2. This decision was made because the enablers and inhibitors listed from each source is comprehensive – it goes beyond the main themes identified in the body of the manuscript. The body of the manuscript summarized the main themes we identified, but each article still has some enablers/inhibitors that do not fit the themes perfectly which is why we wanted to keep them listed in the table. Thank you again. |
| Discussion – “the majority of studies were published after 2017”… would be useful to have a number or % in brackets. | Thank you for bringing this to our attention – it has been updated.  N= 28 studies published 2017 and onwards (page 21, line 20). |
| Discussion – some commentary around overall trends in homeless peer workers would be beneficial. For example, in Australia, while there is wide acceptance of peer workers in MH and AOD, homelessness is still very much in its infancy and there are few examples of where its working well. While there may be some small examples of services that have a peer worker (or two), I know there has been an overall feeling around the general lack of support/guidance, and feeling “of being on their own”, training programs haven’t really taken off here and organisations are resource poor to implement this – which of course can have grave consequences around feelings of re-traumatisation on sharing their experiences and crossing those professional boundaries etc.. Where we know of individuals doing this sort of work we have connected them so they can share experiences/resources etc., but this is all very small scale. | We appreciate your insightful comment. However, in the results (under inhibitors) and in the concluding paragraph, it acknowledges these barriers and the fact that further investigation is needed to understand how peer and lay support work programs are successfully implemented within the homeless population in different contexts in order to build best practice recommendations and to promote success of these programs (i.e., this is a novel concept and work is needed to address these barriers regarding lack of support/guidance, etc.). We would be happy to -do whatever the reviewer suggested- if the editors prefer. |
